# Response to comment on “association between the dietary index for gut microbiota and osteoporosis among middle-aged and older adults in the United States”

**DOI:** 10.1016/j.pmedr.2025.103302

**Published:** 2025-11-06

**Authors:** Yiming Zhan, Yuhang Liu, Jialing Tang, Siyao Gao

**Affiliations:** a Department of Physical Education, Central South University, Changsha 410083, PR China; b School of Physical Education, Wuhan Sports University, Wuhan 430079, PR China

## Abstract

This correspondence is a response to the comment by Dr. Xiaoling Xu on our recently published article, “Association between the dietary index for gut microbiota and osteoporosis among middle-aged and older adults in the United States.” We address the issues raised regarding residual confounding, subgroup analyses, and clinical outcome definitions, and provide clarifications and perspectives for future research.

Dear Editor,

We thank Dr. Xu for the thoughtful and constructive comments on our article, “Association between the dietary index for gut microbiota and osteoporosis among middle-aged and older adults in the United States”. We highly value this academic exchange, which helps refine our interpretation and enhance the translational relevance of our work. Our detailed responses are provided below.

We fully agree with Dr. Xu's thoughtful comments regarding potential residual confounding and data analysis methods. Our study specifically focused on clinically defined osteoporosis, while osteopenia represents an intermediate diagnostic category. Including individuals with osteopenia could have introduced heterogeneity and confounded the conclusions specific to osteoporosis. To ensure clinical consistency and accurate interpretation of the results, we therefore chose to analyze only participants with confirmed osteoporosis(Zhan et al., 2025). This rationale was explained in the Methods section and supported by classic literature. As stated in the article, “To reduce diagnostic heterogeneity and ensure alignment with clinically relevant outcome definitions, participants with osteopenia were also excluded(Looker et al., 1997).”

In addition, we made every effort to minimize potential bias by carefully adjusting for a broad range of covariates based on previous research and clinical relevance. Several potential confounding variables were meticulously evaluated, drawing on both existing literature and clinical expertise. These variables included age, gender, race/ethnicity, marital status, educational level, poverty-income ratio, alcohol consumption, smoking status, BMI, sedentary behavior, sleep duration, total cholesterol, and HDL-C (Hou et al., 2023; Tang et al., 2024). However, we acknowledge that we were unable to control for certain important confounders such as vitamin D status, calcium‑phosphorus metabolism, and physical activity levels and other unmeasured variables. As noted in the Limitations section of our paper, “Second, there may be potential residual confounders due to unmeasured factors or measurement error in the dietary recall data. Third, the DI-GM was calculated from 24-h dietary recalls and may not accurately reflect habitual intake. Future longitudinal studies and randomized controlled trials are necessary to confirm these findings and explore the mechanisms behind them (Zhan et al., 2025).” We fully agree that future studies employing longitudinal designs and more refined methodologies, such as direct measurement of gut microbiota characteristics, will provide stronger evidence.

Regarding the absence of subgroup analyses, we recognize the importance of such analyses in identifying potential effect modifiers. The primary objective of our study was exploratory, focusing on the overall association between the DI-GM and osteoporosis in the general population. Considering this exploratory focus and the sample size limitations, we did not include detailed subgroup analyses in the initial report. Nevertheless, we agree that stratified analyses by sex, race/ethnicity, menopausal status, and other demographic or clinical factors could provide valuable insights. In future work, we plan to examine whether the association between DI-GM and osteoporosis varies across these subgroups, as this may reveal heterogeneity and inform more targeted prevention strategies.

Finally, we acknowledge the clinical importance of FRAX scores and fracture outcomes as endpoints in osteoporosis research. These outcomes are indeed crucial for risk assessment and patient management. In our study, we adopted the classical definition of osteoporosis—femoral neck BMD T-score ≤ −2.5, consistent with the WHO criteria and previous literature (Kanis, 1994). This definition has been widely used for epidemiological classification of osteoporosis. Unfortunately, the NHANES cycles we analyzed did not include data on 10-year FRAX fracture probabilities or longitudinal fracture outcomes; thus, we were unable to incorporate these endpoints. We agree that evaluating DI-GM using validated fracture risk indicators such as FRAX or actual fracture incidence would enhance clinical generalizability. To address this, we plan to conduct future validation studies using independent cohorts and datasets such as the NHANES 2013–2014 wave, the China Health and Retirement Longitudinal Study, and the UK Biobank cohort, where additional outcome data are available. These studies will allow us to test the robustness of the association between DI-GM and osteoporosis, as well as its predictive value for fracture risk across diverse populations, thereby improving the clinical applicability of our findings.

In conclusion, we again express our sincere gratitude to Dr. Xu for her careful review and constructive feedback. Her comments have provided valuable insights that will help us refine future analyses and extend the clinical and public health relevance of our work.

## CRediT authorship contribution statement

**Yiming Zhan:** Writing – original draft, Writing – review & editing. **Yuhang Liu:** Writing – review & editing. **Jialing Tang:** Writing – review & editing. **Siyao Gao:** Validation, Visualization, Writing – review & editing.

## Funding sources

M.Ed. Tang is supported by grant 22YJC890029, the (10.13039/501100002701MOE Ministry of Education in China) Project of Humanities and Social Sciences. The funding bodies had no roles in the design of the study and collection, analysis, and interpretation of data, and in writing the manuscript.

## Declaration of competing interest

The authors declare that they have no known competing financial interests or personal relationships that could have appeared to influence the work reported in this paper.

## Data Availability

No data was used for the research described in the article.
